# Early Treatment of Interleukin-33 can Attenuate Lupus Development in Young NZB/W F1 Mice

**DOI:** 10.3390/cells9112448

**Published:** 2020-11-10

**Authors:** Fatin Nurizzati Mohd Jaya, Zhongyi Liu, Godfrey Chi-Fung Chan

**Affiliations:** Department of Pediatrics and Adolescent Medicine, Faculty of Medicine, The University of Hong Kong, 21 Sassoon Road, Hong Kong, China; fatinjay@hku.hk (F.N.M.J.); liuzhy@connect.hku.hk (Z.L.)

**Keywords:** Interleukin-33, cytokine, systemic lupus erythematosus, regulatory B cells, autoimmune diseases

## Abstract

Interleukin-33 (IL-33), a member of the IL-1 cytokine family, has been recently associated with the development of autoimmune diseases, including systemic lupus erythematosus (SLE). IL-33 is an alarmin and a pleiotropic cytokine that affects various types of immune cells via binding to its receptor, ST2. In this study, we determine the impact of intraperitoneal IL-33 treatments in young lupus, NZB/W F1 mice. Mice were treated from the age of 6 to 11 weeks. We then assessed the proteinuria level, renal damage, survival rate, and anti-dsDNA antibodies. The induction of regulatory B (Breg) cells, changes in the level of autoantibodies, and gene expression were also examined. In comparison to the control group, young NZB/W F1 mice administered with IL-33 had a better survival rate as well as reduced proteinuria level and lupus nephritis. IL-33 treatments significantly increased the level of IgM anti-dsDNA antibodies, IL-10 expressing Breg cells, and alternatively-induced M2 macrophage gene signatures. These results imply that IL-33 exhibits a regulatory role during lupus onset via the expansion of protective IgM anti-dsDNA as well as regulatory cells such as Breg cells and M2 macrophages.

## 1. Introduction

Interleukin-33 (IL-33) belongs to the inflammatory IL-1 cytokine family [1]. Constitutive expression of IL-33 can be found in various organs, including the lung, spleen, kidney, brain, and heart [1,2]. Higher expression of IL-33 has been reported in the barrier tissues, such as the epithelial and endothelial presumably due to its role as a tissue-derived alarmin and damage-associated molecular pattern (DAMP) of the immune system [1]. Upon tissue damage and necrosis, the active full length of IL-33 is released and signals through a heterodimeric receptor complex, ST2, and interleukin-1 receptor accessory protein (IL-1RAcP) [3]. IL-33 signaling leads to the downstream activation of nuclear factor (NK)-κB and increased production of T-helper 2 (Th2) cytokines such as IL-4, IL-5, and IL-13 [4,5]. IL-33 can act on numerous immune cells including T cells (Tregs), T helper (Th) 2 cells, mast cells, and type 2 innate lymphoid (ILC2s) cells [6,7]. Due to alternative splicing, extracellular soluble ST2 (sST2) can be formed as a decoy receptor and limit IL-33 activity [8]. As a pleiotropic cytokine, the involvement of IL-33 in autoimmune diseases such as in rheumatoid arthritis (RA), inflammatory bowel diseases (IBD), and systemic lupus erythematosus (SLE) has been described [9].

SLE is a chronic inflammatory disorder that severely affects multiple organs. SLE pathogenesis involves a complex interplay of genetic, immunological, and environmental factors, leading to aberrant innate and adaptive immunity. SLE presents various clinical manifestations including high production of autoantibodies and inflammatory cytokines [10].

Increasing evidence suggests the vital role of IL-33 in the development of SLE. Increased levels of soluble ST2 protein was reported in SLE patients with active disease as compared to patients with inactive disease and healthy individuals [11,12]. Blocking IL-33 activity in lupus MRL/*lpr* mice resulted in disease improvement [13]. Moreover, a greater level of serum IL-33 was reported in Chinese SLE patients as compared to healthy individuals, albeit lower than in patients with RA [11]. Additionally, a case-study performed in the Chinese population reported an association between IL-33 polymorphism and susceptibility to SLE [14,15]. In contrast, a contradictory finding has been reported by Mok et al. in which the serum level of IL-33 in SLE patients was not correlated to disease severity [12]. This may partially be due to the more dominant role of IL-33 during disease initiation, rather than the late phase. IL-33 was demonstrated to positively correlate to acute-phase inflammatory markers such as C-reactive protein (CRP) and erythrocyte sedimentation rate (ESR), but no correlation to disease course was found [11]. A similar finding was reported by Guo et al., further supporting the crucial role of IL-33 in the early phase of SLE [16]. Therefore, given the considerable evidence pointing to the association of IL-33 in SLE, particularly during the early stage of the disease, it is necessary to further investigate its roles and mechanisms. Moreover, as the role of IL-33 has only been studied in MRL/*lpr* mice, the use of other lupus animal models can verify our postulation.

In the present study, we examined the biologic effect of IL-33 in young lupus-prone NZB/W F1 mice. We found that the administration of IL-33 starting from the age of 6 weeks significantly reduced proteinuria level and mortality rate. Furthermore, IL-33 substantially reduced IgG deposition and renal damage. IL-33 treatments also promoted IgM anti-dsDNA antibodies, IL-10^+^ regulatory B (Breg) cells, and M2 macrophage-associated genes. These data suggest that IL-33 may exert beneficial roles during the early development of SLE.

## 2. Materials and Methods

### 2.1. Ethics Approval

All animal experiments in this study were performed under the guidelines approved by the Committee on the Use of Live Animals and Teaching and Research (CULATR), The University of Hong Kong (HKU) (CULATR 4318-17, approved on 30 March 2017). Licenses specifying experiments involved in this study were obtained from the Department of Health, Hong Kong.

### 2.2. Animals and Treatment

NZB/W F1 mice were bred from the parental strains, NZW and NZB mice at the animal facility, Faculty of Medicine, HKU. Female mice were randomly sorted into two groups and given intraperitoneal (i.p.) injections with 100 μL of 0.4 μg recombinant murine IL-33 (BioLegend, San Diego, CA, USA) or only phosphate-buffered saline (PBS). Treatments were performed twice per week, for five consecutive weeks, from six weeks of age. The treatment protocol was based on an earlier study of IL-33 in colitis model [17].

### 2.3. Measurement of Proteinuria and Anti-dsDNA Antibodies

Beginning at the age of 6 weeks, mice were monitored for proteinuria and body weight once a week. Proteinuria was measured by urine dipstick (Albustix, Bayer). Protein concentration was analyzed semi-quantitatively according to manufacturer’s instruction, as grade 0 (negative), grade 1+ (≥30 mg/dL), grade 2+ (≥100 mg/dL), grade 3+ (≥300 mg/dL), or grade 4+ (≥2000 mg/dL). The serum concentration of anti-dsDNA antibodies was performed as described in [18]. Briefly, serum samples were prepared by diluting 1:500 in PBS containing 1% of bovine serum albumin (BSA). Isotype-specific detection antibodies for murine IgG, IgG1, IgG2a, IgG2b, and IgG3 conjugated with horseradish peroxidase (HRP) (Thermo Fisher Scientific, Waltham, MA, USA) were used at optimum concentrations. For the detection of IgE and IgM anti-dsDNA, anti-IgM and anti-IgE antibodies (BioLegend, San Diego, CA, USA) conjugated with biotin were used before incubation with HRP-conjugated streptavidin.

### 2.4. Renal Histology

For immunofluorescence staining, kidneys were snap-frozen in liquid nitrogen and stored at −20 °C until subsequent experiments. Kidney cryosections were fixed in ice-cold acetone for 5 min and air-dried. Sections were washed and blocked with 10% normal goat serum for one hr. Subsequently, the sections were stained with Alexa Fluor^®^ 488 goat anti-mouse IgG (BioLegend, San Diego, CA, USA) for 1 h at room temperature (RT). Tissue sections were mounted and examined by a conventional fluorescence microscope. The cell fluorescent intensity of IgG deposition in the sections was analyzed using the Image J software (National Institutes of Health, Bethesda, Maryland, USA). For pathological analysis, kidney tissues were fixed in 4% paraformaldehyde solution (PFA), processed into paraffin sections, and stained with hematoxylin and eosin (H&E) by Histopathology Services, Department of Pathology, HKU. Renal histologic abnormalities including glomerular, tubular, and vascular changes were assessed semi-quantitatively by two independent observers in a blinded manner. Scores were classified as 0 (no damage), 1 (mild damage, <25%), 2 (moderate damage, 25–50%), 3 (marked damage, >50–75%) or 4 (severe damage >75% of the total area) [19,20]. At least 30 glomeruli were examined per mouse.

### 2.5. Flow Cytometry Analysis

Weekly blood collection from the lateral saphenous vein was performed during the course of IL-33 and PBS treatments. The lysis of red blood cells (RBC) was performed with a working concentration of RBC lysis buffer (eBioscience, Waltham, MA, USA) according to the manufacturer’s instruction. Cells were stained with Phycoerythrin-Cy7 (PE-Cy7) anti-mouse CD19 (BioLegend, San Diego, CA, USA) for 15 min at 4 °C. For IL-10 intracellular staining, cells were re-stimulated by adding 50 ng/mL phorbol myristate acetate (PMA) (Sigma-Aldrich, St. Louis, MO, USA), 500 ng/mL ionomycin (Sigma-Aldrich, St. Louis, MO, USA), and brefeldin A (BD Biosciences, Franklin Lakes, NJ, USA) for 4 hr. Next, cells were fixed and permeabilized using Cytofix/Cytoperm Kit (BD Biosciences, Franklin Lakes, NJ, USA) according to the manufacturer’s guideline. Cells were then incubated with allophycocyanin (APC) anti-mouse IL-10 for 30 min at RT and washed with Cytoperm buffer before FACS acquisition. Either appropriate isotype controls or unstained samples were used for the gate-setting of cytokine expression.

### 2.6. RNA Extraction and Sequencing Analysis

At week 2 of treatments, whole blood was obtained from PBS-treated and IL-33-treated NZB/W F1 mice. The extraction of total RNA from blood samples was performed with the RiboPure™-Blood RNA Isolation Kit (Applied Biosystems^®^, Thermo Fisher Scientific, Waltham, MA, USA) following the manufacturer’s instructions. Subsequently, alpha- and beta-globin mRNA was depleted from the isolated total RNA using the Globin Clear-Mouse/Rat Kit (Applied Biosystems^®^, Thermo Fisher Scientific, Waltham, MA, USA). RNA sequencing was performed by Novogene (Beijing, China) and library preparation for sequencing was according to standard Illumina protocols. cDNA libraries were sequenced by the Illumina Hiseq 2500 device. For data analysis, the main protocol [21] related to HISAT2 [22], StringTie [23], and Ballgown [24] was followed.

### 2.7. Statistical Analysis

The significant differences between experimental groups were assessed using Student’s t-test or analysis of variance (ANOVA). The survival of animals was presented in Kaplan–Meier curves and compared with the log-rank test. Statistical tests were evaluated with GraphPad PRISM V3 (Graphpad Software, San Diego, CA, USA). *p* values were indicated as * for *p* < 0.05; ** for *p* < 0.01; and *** for *p* < 0.001.

## 3. Results

### 3.1. Early IL-33 Treatments Reduced Proteinuria Level and Prolonged Survival in NZB/W F1 Mice

**Figure 1:**
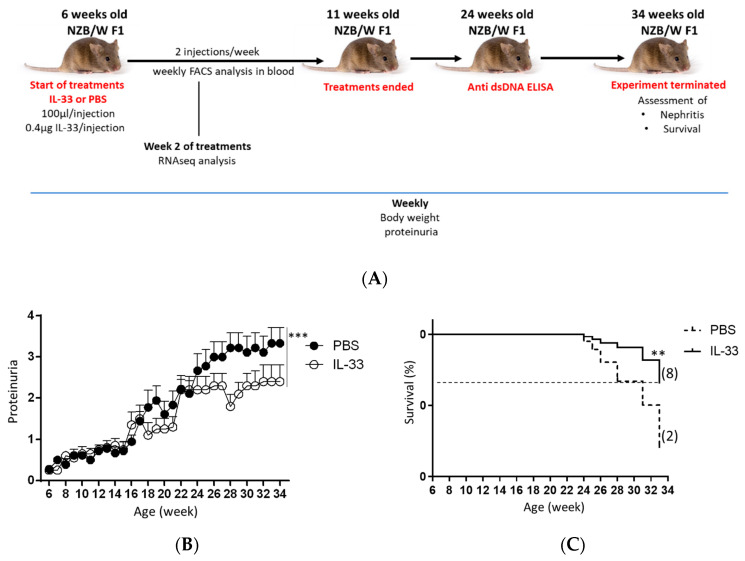
Suppressed proteinuria level and mortality rate in NZB/W F1 mice treated with interleukin-33 (IL-33). Beginning at 6 weeks of age, NZB/W F1 mice were given peritoneal treatments of IL-33 or PBS, two times a week for five continuous weeks. (**A**) Experimental outline. (**B**) Proteinuria was measured bi-weekly and graded as 0+, 1+, 2+, 3+, or 4+. Values represent the mean ± SEM. *** *p*  <  0.001, analyzed by two-way ANOVA. (**C**) Survival rate (percentage) showing mice that had to be sacrificed due to severe lupus (more than 3+ proteinuria and 20% loss of body weight). Kaplan–Meier survival curves, *n* = 12, analyzed by log-rank test, ** *p*  <  0.01. The numbers of mice surviving to 33 weeks of age are shown in parentheses. Results represent two independent experiments.

Female NZB/W F1 mice develop disease from 4 to 6 months of age [25]. To evaluate the effect of IL-33 on the onset of disease, the effect of IL-33 on lupus development was analyzed by treating 6-week-old NZB/W F1 mice with IL-33. Mice treated with PBS solution served as the control group. First, the level of proteinuria in the two experimental groups was assessed bi-weekly from the beginning until the termination of experiment (34 weeks of age) (Figure 1A). We found that early administration of IL-33 substantially suppressed the development of proteinuria (*p* < 0.001, Figure 1B). Further analysis revealed that mice treated with IL-33 before the manifestation of symptoms had a prolonged life span as compared to the control group. The difference in survival rate between IL-33-treated and PBS-treated groups was found to be significant (*p* < 0.01, Figure 1C). These results suggest that the early treatment of IL-33 starting from the age of 6 weeks helps to delay disease onset in NZB/W F1 mice.

### 3.2. Early IL-33 Treatments Reduced IgG-Immune-Complex Deposition and Glomerular and Tubular Damage in NZB/W F1 Mice

**Figure 2:**
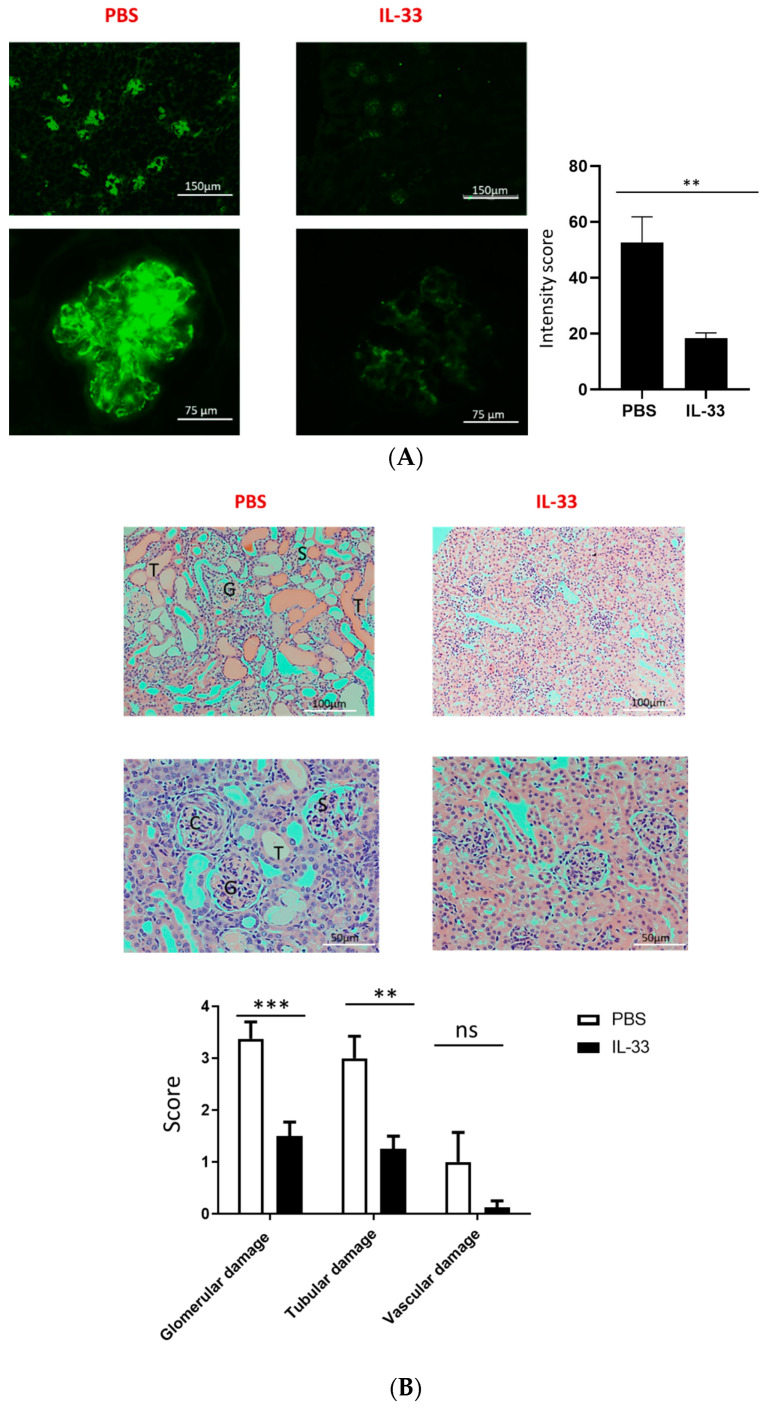
Reduced IgG deposition in the kidney of NZB/W F1 mice treated with IL-33. Beginning at 6 weeks of age, NZB/W F1 mice were given peritoneal treatments of IL-33 or PBS, two times a week for five continuous weeks. (**A**) Representative samples of IgG deposition in the kidney from PBS- (left panel) and IL-33- (right panel) treated 34-week-old NZB/W F1 mice as visualized by immunofluorescence staining. Original magnification 100× (upper panel) and 200× (lower panel). Less IgG deposition was detected in the IL-33-treated mice. Comparison in the intensity score as measured using ImageJ software. *n* = 8. (**B**) Representative samples of H&E staining in the kidney from PBS- (left panel) and IL-33- (right panel) treated 34-week-old NZB/W F1 mice. Glomerular, tubular, and vascular damages were scored from 0 to 4+. G, glomerulonephritis with fibrosis; C, crescentic glomeruli; S, sclerotic glomeruli; and T, tubular dilation with casts. Original magnification 200× (upper panel) and 400× (lower panel). *n* = 10. Values represent the mean ± SEM. ** *p* < 0.01, *** *p* < 0.001, analyzed by Student’s t-test. Result represents at least two independent experiments.

One of the hallmarks of lupus pathogenesis is the deposition of antibody complex in the kidney. We then analyzed the deposition of IgG in the glomeruli of IL-33- and PBS-treated mice by immunofluorescence microscopy at the age of 34 weeks. The fluorescence intensity was assessed, and we found that IL-33-treated mice displayed a substantial reduction in glomerular staining of IgG as compared to the control mice (Figure 2A). In addition to the immune-complex deposition, histologic evaluation on the glomerular, tubular, and vascular damage was also performed with H&E staining. Mice receiving IL-33 injections had a significant decrease in glomerular and tubular damage scores (Figure 2B). However, no significant differences were observed in vascular damage.

### 3.3. Early IL-33 Treatments Induced IgM Anti-dsDNA Antibodies in NZB/W F1 Mice

**Figure 3:**
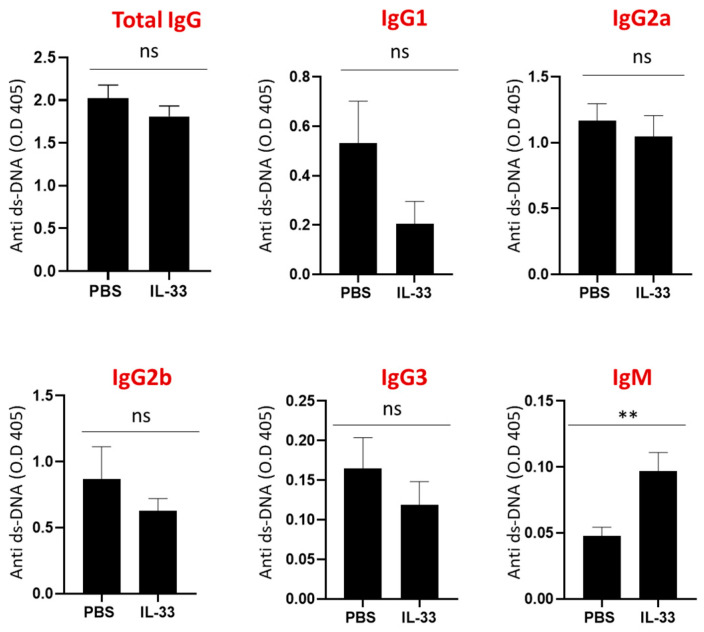
Increased serum IgM anti-dsDNA in NZB/W F1 mice treated with IL-33. Beginning at 6 weeks of age, NZB/W F1 mice were given peritoneal treatments of IL-33 or PBS for five weeks. At the age of 24 weeks, serum samples were collected for the measurement of anti-dsDNA antibodies by ELISA. No significant changes in the concentration of total IgG, IgG subclasses, or IgE anti-dsDNA levels were detected. Significant increase of IgM anti-dsDNA level by IL-33 was detected. Values represent the mean ± SEM. Ns, not significant; ** *p*  <  0.01, analyzed by Student’s t-test. *n* = 7, Result represents at least two independent experiments.

Next, the serum levels of IgG subclasses, IgM, and IgE anti-dsDNA antibodies were determined at the age of 24 weeks. No significant differences were detected in the IgG subclasses and IgE anti-dsDNA antibodies between IL-33- and PBS-treated groups (Figure 3). Interestingly, mice that received IL-33 treatments displayed a higher level of IgM anti-dsDNA antibodies (*p* < 0.01). Some key experiments have demonstrated the protective roles of IgM anti-dsDNA antibodies against the development of SLE and lupus nephritis [26,27]. Thus, these results suggest that IL-33 regulates disease activity through the induction of IgM anti-dsDNA antibodies.

### 3.4. IL-33 Treatments Increased the Peripheral Breg Cells in NZB/W F1 Mice

**Figure 4:**
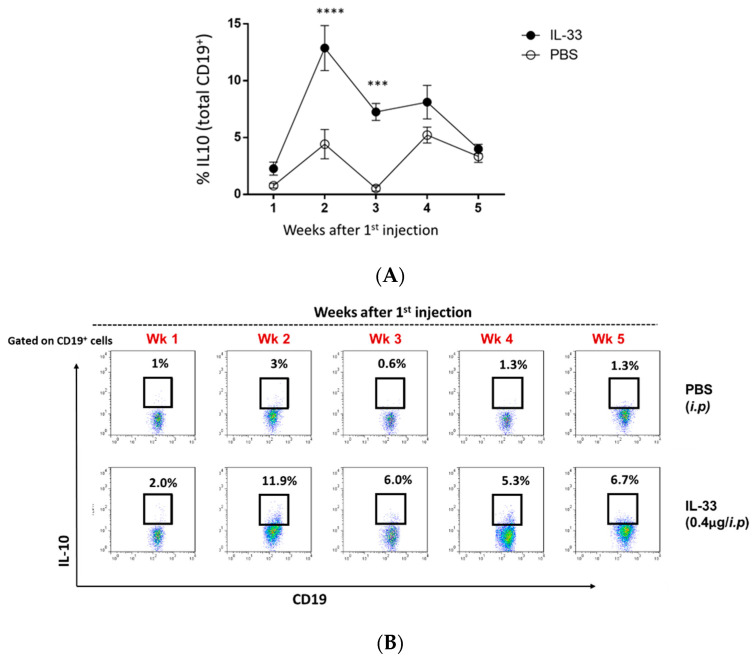
IL-33 increased the frequency of circulating IL-10^+^ regulatory B (Breg) cells in young NZB/F1 mice. Beginning at 6 weeks of age, NZB/W F1 mice were given peritoneal treatments of IL-33 or PBS, two times a week for five continuous weeks. Throughout the treatments, weekly blood samples were obtained and re-stimulated with PMA/ionomycin/brefeldin for the detection of IL-10-expressing CD19^+^ cells with flow cytometry. (**A**) Representative plots of IL-10^+^ Breg cells in the PBS- and IL-33-treated mice. (**B**) Comparison of the percentage of Breg cells. *n* = 12, bars represent the mean ± SEM. *** *p* < 0.001, **** *p*  <  0.0001, two-way ANOVA with multiple comparisons were performed. Results represent three independent experiments.

It was previously shown that IL-33 could induce peripheral Breg cells in the wild-type C57BL/6 mice [17]. Therefore, we investigated the induction of Breg cells in the circulation throughout PBS/IL-33 injections, from the age of 6 to 11 weeks. Blood samples were obtained and stained for CD3, CD4, FOXP3, CD19, and IL-10. For the detection of intracellular IL-10, cells were re-stimulated with PMA, ionomycin, and brefeldin for 4 hr. As expected, it was found that IL-33 treatments significantly induced the expansion of IL-10^+^ Breg cells in young NZB/W F1 mice (Figure 4A,B). The induction of Breg cells was observed to be the highest during week 2 of IL-33 injections (Figure 4A,B). In contrast, minimal changes in the level of Breg cells were detected in the PBS-treated group. Additionally, no significant changes were detected in other analyzed subsets (S1). Thus, this suggests that the beneficial effect of IL-33 treatments was mediated by Breg cells.

### 3.5. IL-33 Treatments Induced M2-Macrophage-Related Genes in NZB/W F1 Mice

**Figure 5:**
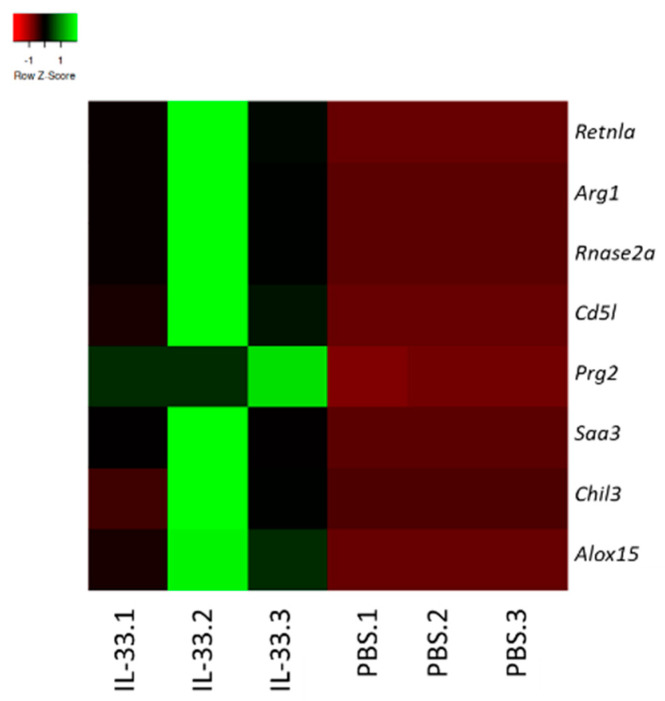
IL-33 treatments upregulated M2-related immunosuppressive genes. Total RNA was extracted from peripheral blood of NZB/W F1 mice at week 2 of IL-33/PBS treatments. Heatmap of genes showing differential expression in the IL-33 and PBS group. Genes were filtered for moderate to high expression and fold change > 2. Green is high expression, red is low expression, and black is intermediate. *n* = 3. *Retnla*, resistin-like molecule alpha/FIZZ1; *ARG1*, arginase 1; *Rnase2a*, ribonuclease A family member 2; *CD5L*, CD5-molecule-like; *Prg2*, proteoglycan 2; *Saa3*, serum amyloid A3; *Chil3*, chitinase-like 3; *Alox15*, arachidonate 15 lipoxygenase.

To determine the possible impact of IL-33 treatments on the transcriptome of young NZB/W F1 mice, RNA was extracted from whole blood and subjected to RNA-seq analysis. We analyzed the gene expression patterns in the samples obtained from PBS- and IL-33-treated groups. The top genes that were upregulated upon IL-33 administration were resistin-like molecule alpha *(retnla)/Fizz1*, arginase 1 (*Arg1)*, ribonuclease A family member 2 (*rnase2*), CD5-molecule-like (*CD5L*), proteoglycan 2 (*prg2*), serum amyloid A3 (*saa3*), chitinase-like 3 (*chil3*)*,* and arachidonate 15 lipoxygenase (*alox15*) (Figure 5). Interestingly, these genes have been widely implicated in the development of the suppressive, alternatively-activated M2 macrophages [28,29,30,31,32]. Therefore, this finding implicates that IL-33 may contribute to the resolution of inflammation, partly by upregulating M2-associated markers and polarization.

## 4. Discussion

Although the involvement of IL-33 in SLE has been shown in multiple animal and clinical researches, its role in SLE remains elusive. Furthermore, its effect on NZB/W F1 lupus mice has not been investigated. In the current study, we demonstrated that early IL-33 treatments improved SLE manifestations in young NZB/W F1 mice. This effect was associated with the induction of Breg cells, IgM anti-dsDNA antibodies, and M2 macrophage gene signatures.

IL-33 may exert its protective action in young NZB/W F1 mice via several mechanisms. Numbers of studies have proposed the protective capacity of IgM anti-dsDNA autoantibodies for glomerulonephritis and immune complex-mediated tissue damages [27,33,34]. IgM anti-dsDNA antibodies have been negatively correlated with the development of glomerulonephritis [33]. Furthermore, transfer of anti-dsDNA monoclonal IgM to lupus mice delayed proteinuria development and prevented immune-complex deposition [26,27]. Here, we demonstrated that IL-33 substantially increased the level of IgM anti-dsDNA in NZB/W F1 mice. This finding implies that the disease-suppressive role of IL-33 was partly through the increase of IgM anti-dsDNA antibodies. However, further study is required to confirm the protective role of IgM anti-dsDNA antibodies induced by IL-33 in NZB/W F1 mice. Additionally, IL-33 may also limit kidney injury via an unknown mechanism independently from IgM-anti dsDNA antibodies. Whether IL-33 could directly affect immune or non-immune renal cells such as the podocytes, distal or proximal tubular cells, awaits further investigation.

An alternative explanation of the protective effect of IL-33 is via its regulatory role in inducing Breg cells. Here, we found that IL-33 treatments significantly increased the frequency of IL-10^+^ Breg cells in the peripheral blood. This is consistent with several reports on the impact of IL-33 in inducing Breg cells in other animal models [17,35]. The beneficial role of Breg cells in suppressing inflammation and limiting the development of autoimmune diseases, including SLE, has been known [17,36,37]. For example, CD1d^hi^CD5^+^ Breg cells contributed to the prolonged survival and suppressed lupus progression in NZB/W F1 mice [38].

Apart from promoting the frequency of Breg cells, RNA sequencing analysis revealed that IL-33 treatments upregulated M2-associated genes. As opposed to the M1 macrophages, M2 macrophages are important in the resolution of inflammation, maintenance of self-tolerance, and tissue healing [7]. Adoptive transfer of M2 macrophages remarkably attenuated SLE severity [7]. Although we did not show the actual increase of M2 macrophages in our study, nonetheless, this finding is in accordance with other reports demonstrating the potent effect of IL-33 in skewing M2 macrophages [39,40]. Of note, it would be interesting to study the therapeutic impact of IL-33-induced M2 macrophages in lupus.

As opposed to our finding, IL-33 has been suggested to exhibit a disease-promoting role in the MRL/*lpr* lupus mice [13]. Li et al. demonstrated that blocking IL-33 using monoclonal antibody from the age of 14 to 20 weeks, protected MRL/*lpr* mice from lupus development through inhibition of Th17 cells and expansion of Tregs. The difference in our findings may be caused by different factors such as types of animal models adopted, dosage, and possible multi-faceted functions of IL-33. In comparison to NZB/W F1 mice, MRL/*lpr* mice have different genetic backgrounds and exhibit spontaneous *lpr* mutation which renders them susceptible to developing massive lymphadenopathy, cutaneous lesions, and accelerated disease. This finding, therefore, warrants further study to assess the role of IL-33, if any, in different genetic backgrounds.

The conflicting findings may also be due to the possible paradoxical roles of IL-33 in different immune subsets and disease stages [41]. In SLE patients, IL-33 level was significantly correlated to acute pro-inflammatory biomarkers suggesting that IL-33 is more involved during early disease initiation [11]. Furthermore, during early development of disease, IL-33 may be released from damaged cells as an ‘alarmin’, acting as a signal of ‘danger’ to the immune system [42]. As we have demonstrated in this study that IL-33 could promote a significant portion of Breg cells, it is tempting to speculate that the disease-protecting effect of IL-33 in young NZB/W F1 mice is mediated by this subset. It has been suggested that B cells exert crucial regulatory roles during the early rather than late disease phase [43]. This is also based on an earlier study performed by Yanaba et al. in which early B cell depletion before collagen immunization substantially improved disease activity in a collagen-induced arthritis model [44]. On the other hand, blocking B cells after collagen immunization failed to show any remarkable changes in arthritis severity [44]. Therefore, the disease-suppressing effects of IL-33 in young mice may be due to the regulatory properties of Breg cells induced during disease perpetuation. However, as the disease progresses, excessive signals from IL-33 may otherwise lead to unwanted pathogenic responses such as promoting autoantibody production and inflammation. More studies in immune cell type-specific knockouts at various disease stages will better define the role of IL-33 in the pathogenesis of SLE.

IL-33 has been described as a pleiotropic cytokine with a wide range of actions on the immune system. Conflicting actions of IL-33 have also been described in other models of autoimmune diseases, such as experimental autoimmune encephalomyelitis (EAE) [45,46,47]. EAE mice deficient in IL-33 receptor displayed a rapid progression of disease suggesting protective roles of IL-33 [45]. IL-33 treatment also resulted in a significant improvement of disease [45]. On the other hand, treatment with anti-IL-33 antibody significantly suppressed disease activity and production of IL-17, suggesting its pathogenic roles [46]. Moreover, the seemingly dual action of IL-33 has also been shown in non-autoimmune models [48]. In cardiovascular disease models, IL-33 was demonstrated to promote tissue repair and protect against inflammation via expansion of Treg and ILC2 [48,49]. In contrast, some studies have reported the proinflammatory property of IL-33 in driving angiogenesis and tissue damage via stimulation of mast cells [50].

Increasing evidence supports the conclusion that IL-33/ST2 signaling is involved in the pathophysiology of SLE and other autoimmune diseases. However, the exact role of IL-33 seems to be more complex and likely operates as a double-edged sword in suppressing or promoting inflammation. The role of IL-33 extends beyond its function as the amplifier of inflammation. Hence, to explore the translational potential of IL-33, it is essential to understand the factors associated with its detrimental vs. beneficial effects. A more targeted approach may be necessary to fully understand the dynamic actions of this cytokine prior to actual clinical application. Moreover, the ability to use IL-33 to manipulate and expand Breg cells ex-vivo opens a wide opportunity for clinical application for autoimmune and inflammatory diseases. A very important caveat in our study is that the protective action of IL-33 was only shown in young mice with early signs of disease manifestation. Therefore, it is vital to conduct a similar study in older mice. Nevertheless, this study provides new evidence of the previously-unexplored disease-protecting roles mediated by IL-33 in delaying lupus onset.

## 5. Conclusions

Overall, the findings in this study revealed the novel effect of IL-33 in suppressing lupus onset in young NZB/W F1 mice, presumably through the protective anti-dsDNA IgM antibodies, IL-10-expressing Breg cells, and upregulation of alternatively-activated M2 macrophage-genes.
